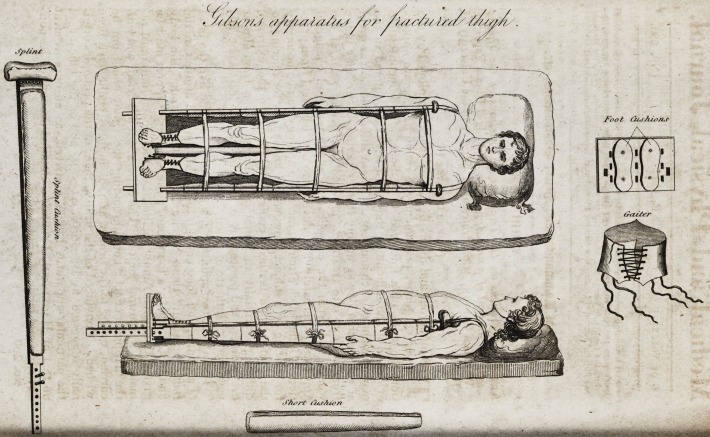# Reflections on the Treatment of Fractures of the Thigh; with an Account of a New Apparatus

**Published:** 1822-06

**Authors:** William Gibson

**Affiliations:** Professor of Surgery in the University of Pennsylvania.


THE LONDON
### Medical and Physical Journal.

6 OF VOL. XLVII.] JUNE, 1822. [No. 280.

For many fortunate discoveries in medicine, and for the detection of numerous errors, the world is indebted to the rapid circulation of Monthly Journals; and there never existed any work, to which the Faculty, in Europe and America, were under deeper obligations, than to the Medical and Physical Journal of London, now forming a long, but an invaluable, series.—RUSH.

### Original Communications, Select Observations, etc.

### CHIRURGERY.

**Art. I.**—*Reflections on the Treatment of Fractures of the Thigh; with an Account of a new Apparatus.* Abridged from a Paper by William Gibson, m.d. Professor of Surgery in the University of Pennsylvania.

[With an Engraving.]

THE numerous and diversified contrivances employed by the older surgeons, permanently to retain in accurate apposition the ends of a fractured thigh-bone, afford incontestible evidence of the difficulties they must have encountered in the management of such accidents. These difficulties are as great at the present day as at any former period, if we may judge from the opposite treatment pursued in different parts of the world, and from the complicated, expensive, and changeful apparatus, alternately introduced into and banished from practice. Under such circumstances, no apology can be necessary for treating of a subject, however hackneyed, or for offering to the profession a method, confessedly simple in the extreme, differing essentially in principle and application from almost every other, and possessing, it is believed, at least some advantages not hitherto attained.

I construct two splints, each padded or stuffed like the head of a crutch, and long enough to reach from the arm-pit to the foot, and secure these by circular bandages around the body and limbs, and by a foot-board; the necessary support must be given, the pelvis cannot incline, and the broken limb must remain of its natural length. The experiment was tried, and with the happiest effect; notwithstanding the splint could not be carried, owing to the broken arm, as high as could have been wished. Convinced of the utility, then, of the principles originally suggested by Brunninghausen, and of the efficacy of the particular apparatus I have contrived, as an improvement on that of Hagedorn, I submit a detailed account of its construction and mode of application.

Two splints, half an inch thick, formed at the upper extremities like the head of a crutch, five inches wide immediately below this head, five feet and a half in length, and tapering towards the lower end, which is about two inches wide, constitute a sort of enclosure for the body and limbs, from the arm-pits beyond the feet. The lower end of each splint, to the extent of a foot, is straight, and has six or eight holes, at equal distances, large enough to receive a stout peg, intended to secure the foot-board. Shoulders, also, are made in the splint, just above the upper peg-hole, for the purpose of preventing the foot-board from ascending. The foot-board itself is made of seasoned tough wood, is an inch thick, about twelve inches long and nine high. At different distances in it, there are three rows of slits, half an inch wide and an inch and a half long, intended for the gaiter straps or bandages which secure the feet to the board. Two other slits or mortices, of the same kind, receive the lower ends of the splints; making, in all, eleven perforations through the foot-board. The gaiters are made of soft leather lined with buckskin, or of strong linen, well quilted on the inner surface; are laced to the leg above the ancle by a cord; and have four straps to each, two near the instep, and two near the heel, sufficiently long to pass through the foot-board and admit of being tied on its back part. Two cushions or junks, made of old linen, the breadth and length of the splints, and an inch thick; together with another cushion of the same kind, and long enough to reach from the perinæum to the foot; a splint or junk cloth, similar to that of Dessault, wide enough to enrol the splints several times, and long enough to extend from the perinæum to the ancle; several tapes or pieces of roller; constitute the remainder of the apparatus.*

Antecedent to the adjustment of the limb, and the application of the splints and bandages, a bedstead should be selected, with a board bottom, and over this be placed a thick and firm mattress. If a feather-bed be employed, it will be quite impossible to prevent deformity and inconveniences, however well contrived the apparatus may be. This is a point, therefore, which should always be insisted on by the surgeon; and, if no mattress be at hand, blankets or quilts must be substituted, and laid over the bedstead or floor. The mattress being covered by sheet, the first step on the part of the surgeon is to take six or eight tapes, or pieces of common roller, three or four feet long, and arrange them transversely at different distances, from the foot towards the head of the bedstead. The splint cloth is next laid over the tapes, its longest diameter running parallel with

* To prevent the patient from removing his arm-pit from the crutch-like head of the splint, a strap is fastened to it and crosses the shoulder.

them. The patient is now placed on the mattress, his clothes being previously stripped off, or cut away, as occasion may require; his body is kept perfectly straight; and both limbs placed as accurately as possible over the centre of the dressings. Extension and counter-extension being made, and the ends of the bones coaptated, the splints, previously covered by their cushions, which are placed on the inner sides, and serve to take off any unpleasant pressure, are next rolled up in the splint cloth, and brought closely in contact with the body and limbs. The surgeon next fixes the gaiters to the ancle, and fastens the foot-board to the splints. The feet, having two small cushions beneath them to rest on, are next secured to the foot-board by passing the straps through the holes, and tying them on the outside. All that remains is, fix the third cushion or strap between the thighs, to pass the tapes around the limbs, splints, and body, and secure the whole, so as to constitute, as it were, one solid piece.

As all patients, with fractured thighs, experience more or less inconvenience from the difficulty of having a passage, and as this difficulty will exist with any apparatus, however constructed, it becomes very desirable to obviate it as much as possible, by employing such means as are the most simple, and at the same time most effectual. The ingenious contrivance of Mr. Henry Earle,* for suspending the patient, temporarily, on a strong canvass or sacking bottom, stretched upon a frame, and raised by pulleys or a jack, however well adapted to an hospital or any large establishment, cannot, from its complex and expensive structure, be introduced into private practice, even in a large city, much less in country places. Fortunately, however, every possible advantage may be derived by using Mr. Earle's principle, together with the most simple but effectual part of his machine. For several years past I have used a common frame, seven feet long and three feet wide, upon which is tacked a sacking bottom, having a hole in its centre about the size of the crown of a hat. The sacking bottom is supported by girths passed beneath it, and secured to the frame. As this simple apparatus can be adapted to any common bedstead, it may be kept constantly on hand by the surgeon, or manufactured, at a few minutes' notice, if required, by the most common mechanic in any country place. When prepared, it will be only necessary to lay the frame over the mattress, place the dressings on the sacking bottom, (previously covered by a sheet with a hole in it corresponding to the one in the sacking bottom,) and the patient on the dressings, taking care that his buttocks be exactly adapted to the opening. Upon this very simple contriv-

---

* See Pott's Works, by Sir James Earle, vol. i.

ance, the patient will lay with as much comfort as on the mattress itself; and, whenever he desires to have a stool, it will only be necessary for two assistants, one at each end of the bed, to raise the frame six or eight inches from the mattress, and support it in this situation by a small block placed at each corner of the frame, or by a moveable leg or foot permanently attached to the frame. As I have employed this machine upon several occasions, I can speak with confidence of its efficacy, and can recommend it as equal, if not superior, to the original apparatus itself, which I have frequently seen in operation in the European and American hospitals. But, whether this modification of Earle's bedstead be employed or not, I have ascertained, to my satisfaction, that the draw sheet, or dish or pewter pan, articles in common use, in cases of fractured thigh, can with greater facility be employed when the patient's limbs and body are secured by the plan I have ventured to propose than by any other mode. Indeed, so firmly are the limbs supported against each other, and so completely is the body fixed, that the whole may be said to constitute but one piece, and the patient can be turned or raised, and the pan slipped beneath him, without in the slighest degree disturbing the fragments of bone.

It may be well, in the next place, to anticipate such objections as may possibly be brought against the principles and practice I have endeavoured to establish. It will be said, perhaps, that the extended position, above all others, is the most painful and inconvenient to the patient; that spasmodic affections are very apt to occur, particularly upon every attempt of the patient to procure sleep; that the confinement of one limb, as usually practised, is irksome enough; that the confinement of both, and of the body also, must be, if not insupportable, painful in the extreme; that the counter extension, which is exerted entirely on the acetabulum and thigh of the sound side, will cause the limb to swell, or to become so much fatigued as to produce great distress; that the gaiters, confining the feet to the board, will cause ulceration of the ancles; that both limbs, from being kept so long in the extended position, will become stiff or anchylosed; that the inclination of the body and pelvis to the affected side cannot be prevented by the lodgment of the crutch-like end of the splint in the arm-pit, for the scapula is a moveable point, being connected to the body chiefly by muscles, which yield to any impulse communicated to them; and that, therefore, the superior fragment of bone cannot be prevented from descending and riding over the inferior fragment;—that, in cases where both thighs are fractured, the principle upon which the apparatus acts must be destroyed, and, consequently, the practice totally inadmissible.

Let us see how far these objections can bear examination.—That the extended position is more painful, for the first few hours, than the semiflexed one, may, perhaps, be admitted: but it has been proved, beyond all doubt, by Dessault, Boyer, Richerand, Roux, and many other distinguished French and continental surgeons, that the inconvenience is temporary only; that the muscles soon become accustomed to their position, and cease to afford uneasiness; that, on the contrary, in the semi-flexed position, however comfortable the patient may feel for a short time after the accident, yet he soon becomes tired, and would give the world to be permitted to extend the limb.

With regard to the second objection, that spasmodic affections follow the extended more than any other position, it may be remarked, that this is by no means the case; that startings, or involuntary twitchings, annoy the patient exceedingly after all fractures of the limbs, be the position what it may, especially upon the patient's falling into a doze, from which he is often roused by a sudden and violent jerking, and sometimes by a movement of the broken ends. But, admitting these startings to accompany the extended more than any other posture, should this be considered a serious objection, when we find it so easy to subdue them by appropriate remedies, such as opium, blood-letting, low diet, topical applications?

As respects the third objection, that the confinement of one limb is bad enough without the confinement of both, it may be asked, if the limb were extended and merely inclosed in Dessault's or Boyer's apparatus, divested of their extending and counter-extending bands, would the mere posture and confinement be sufficient materially to incommode the patient? On the contrary, has it not been already shown, and even acknowledged by Dessault himself, that the chief inconvenience and distress proceeds from the pressure of these bands, and the consequent ulceration? If position itself, therefore, be attended with no unpleasant consequence, certainly the additional confinement of the sound limb can put the patient to no inconvenience, especially as the use he could make of it, when stretched upon his back and unable to rise, would be so very limited as to contribute but in the slightest degree to his comfort. Again, the patient is necessarily confined to his back, whether he use the apparatus of Dessault or any other. What difference, therefore, can it make to him, whether the splints extend to the arm-pits or not, or whether the body be kept perfectly straight, or be permitted to deviate to the right or to the left? If the system of Dessault be rigidly enforced, or if the splint, as advised by Dr. Physick, be carried to the axilla and there secured, can the patient then raise himself in bed, or can he relieve himself by alternately inclining to either side?

Certainly not; for in either case he is as completely fettered as if lashed to a post. If these views be correct, unquestionably no disadvantage can arise from an additional splint, which does not encumber the patient, but only serves to give him support.

The fourth objection relates to counter-extension, sustained by the acetabulum and head of the thigh-bone of the sound side. Admitting this to produce some uneasiness to the patient, is it not better that it should be borne by a limb uninjured, free from inflammation, and devoid of pain, than that the same force should be sustained by a limb acutely sensible and greatly swollen? But is much force really exerted? Does the patient actually complain of the fatigue and irritation? This I can only answer by stating that, in the case in which I employed the mode I have advised, no such distress was occasioned, except for a very short period, notwithstanding the habit of the patient was gouty, and in other respects unfavourable. Indeed, does it not seem rational that the acetabulum and head of the thigh-bone, in their natural state, accustomed as they are to bear at least one-half of the weight of the superincumbent parts, should be fully able to support and counteract all the efforts of the muscles of a broken thigh, and that, too, without sustaining the slightest inconvenience?

The fifth objection, that the gaiters will produce ulceration of the ancles, may be answered by stating that, if so, we are no worse off than if we employed the apparatus of Dessault; for his extending band being a single handkerchief, or piece of cotton or linen, will become twisted, and produce more irritation than a band well quilted and applied to a broad surface.

With regard to the stiffness which follows from a fractured limb, this will be acknowledged to be of little consequence; for mobility is soon restored upon the patient being able to walk: and, if it even were of consequence, it cannot be prevented, and is equally liable to happen in all cases, and in every position that could be devised; and, if anchylosis were to follow, which is extremely rare, certainly every one must allow that it would be infinitely better, in fractures of the lower extremities, to happen in the extended than in the semiflexed position. In the former case, the limb would at least be preserved of its natural length; in the latter it would be shortened: and, although the patient might possibly in both instances be lame, yet he would be less so with the limb straight than crooked.

That the scapula is, in some measure, a moveable point, cannot be denied; but it must be admitted, at the same time, that its muscles are numerous and powerful, and, as such, would be capable of resisting any common force set against them; especially as, by such application, they would be stimulated to resistance. Surely, if they are sufficiently strong to support, in the erect posture, the weight of a very heavy man by crutches, without the scapulæ being much raised, they will at least serve, and that too without an effort, to sustain the body in the recumbent position, and prevent a descent sufficient to incline the pelvis, or enable the superior fragment to ride upon the inferior! But the fact is that the arm and scapula do not move so readily as some imagine; the clavicle, being attached to the sternum, gives very considerable support to the scapula and arm, independently of the resistance afforded by the muscles themselves.

The last objection, where both thighs are broken, is the only one which applies with any force; and this, it must be acknowledged, is altogether insurmountable. Here it must be obvious that neither the apparatus of Hagedorn, nor the one I have proposed, nor any other founded upon the same principles can act. But how often do we meet with such an accident? Not once in five hundred times. Here, then, we still have hardly an objection to the *general* employment of the means I have advised. If both thighs should be broken, I would use the apparatus of Dessault, as modified by Dr. Physick, in whose hands perfect cures, under all circumstances, have been produced. Let not these, however, be too sure a guide for all other practitioners: let it be remembered that it is the peculiar privilege of genius to accomplish ends without advantages, to overcome difficulties insurmountable to ordinary minds,—and that, in the instance of the illustrious character just mentioned, success is always sure to follow, where success is possible.

## Figures and Tables

**Figure f1:**